# Similar strength recovery but more complications with Bone‐Quadriceps Tendon Autograft compared to Quadriceps Tendon Autograft in anterior cruciate ligament reconstruction in competitive soccer players

**DOI:** 10.1002/jeo2.70324

**Published:** 2025-07-13

**Authors:** Jose Luis Martín Alguacil, Manuel Vides Fernánde, Carolina Fernandez‐Lao, Mario Lozano‐Lozano, Fahed Herbawi, Juan Carlos Monllau

**Affiliations:** ^1^ Department of Orthopaedic Surgery Andalusian Mutuality of Soccer Players and Martín Gómez Clinic Granada Spain; ^2^ Department of Orthopedic Surgery and Trauma Hospiten Estepona Hospital Málaga Spain; ^3^ Department of Physical Therapy, Health Science Faculty University of Granada Granada Spain; ^4^ Instituto de Investigación Biosanitaria ibs.GRANADA Granada Spain; ^5^ “Cuídate” Support Unit for Oncology Patients (UAPO‐Cuíate) Sport and Health University Research Institute (iMUDS) Granada Spain; ^6^ Department of Allied and Applied Medical Sciences Faculty of Medicine and Health Sciences An‐Najah National University Nablus Palestine; ^7^ Department of Orthopaedic Surgery, Hospital del Mar Universitat Autònoma de Barcelona (UAB) Barcelona Spain; ^8^ ICATKnee, Institut Catalá de Traumatologia i Medicina de l'Esport (ICATME), Hospital Universitari Dexeus, UAB Barcelona Spain

**Keywords:** anterior cruciate ligament, Bone‐Quadriceps Tendon Autograft, Quadriceps Tendon Autograft, reconstruction, sports

## Abstract

**Purpose:**

This study aimed to evaluate the clinical and functional outcomes, as well as the associated morbidity, of anterior cruciate ligament (ACL) reconstruction using quadriceps tendon grafts, with and without bone block.

**Methods:**

This prospective cohort study involved 52 competitive Spanish federal soccer players aged 16–40. This study compared the outcomes of ACL reconstruction using Bone‐Quadriceps Tendon Autograft (BQTA) and Quadriceps Tendon Autograft (QTA). Functional, clinical and patient‐reported outcomes were assessed preoperatively and at 3‐, 6‐ and 12‐month postoperative intervals. Both groups followed standardized surgical and rehabilitation protocols. Statistical analyses were performed to identify differences in recovery patterns and morbidity, thereby providing insights into the optimal graft selection tailored to patient‐specific needs and activity levels.

**Results:**

A total of 52 patients with ACL injuries were evaluated. No significant differences were observed in the outcomes at the final follow‐up. Key metrics such as the Lysholm Knee Score (mean difference = 1.2, 95% CI: [−0.8, 3.2], *p* = 0.21), Visual Analogue Scale for pain (mean difference = 0.3, 95% CI: [−0.5, 1.1], *p* = 0.68), peak torque, hamstring/quadriceps ratios and Limb Symmetry Index showed no significant differences between the groups. Notably, 2 patellar fractures and one quadriceps tendon avulsion occurred in the BQTA, highlighting the higher morbidity associated with this particular graft.

**Conclusion:**

Similar functional and clinical results were observed with BQTA and QTA after ACL reconstruction in football players. Major complications were associated with the extraction of the bone block exclusively in the BQTA, which can guide the surgeon in choosing the type of quadriceps graft.

**Level of Evidence:**

Level II.

**Abbreviations**
ACL: anterior cruciate ligament
ANOVA: analysis of variance
BQTA: Bone‐Quadriceps Tendon Autograft
FROMs: functional outcome measures
H/Q ratio: hamstring/quadriceps ratio
LKS: Lysholm Knee Scale
LSI: Limb Symmetry Index
PROMs: patient‐related outcome measures
PT: peak torque
QTA: Quadriceps Tendon Autograft
SLHT: single leg hop test
VAS: Visual Analogue Scale

## INTRODUCTION

The optimal graft choice in anterior cruciate ligament (ACL) reconstruction (ACLR) is still a matter of differing opinions, with no definitive agreement to this date [3, 21, 24]. However, over the past 2 decades, the quadriceps tendon graft has increased in popularity [30]. This rising popularity is due to recent scientific data from biomechanical and clinical studies that advocate its use and, in some cases, outperform patellar tendon and hamstring tendon grafts [12, 26, 29].

The quadriceps tendon autograft (QTA) can be harvested in four distinct ways: with or without a bone block and, in each case, either as a full‐thickness or partial‐thickness graft [31]. By including a bone block, the graft may ease the integration in the bone tunnels made during the procedure, which in turn could favour a faster maturation of the graft [18]. However, this method has the drawback of increased morbidity owing to the risk of fracturing the patella [9]. On the other hand, when harvesting a partial‐thickness soft tissue graft, the deeper fibres of the vastus intermedius of the quadriceps tendon are spared, which lowers the morbidity of the donor site as well as fluid extravasation during the surgical procedure.

Recent scientific literature usually concludes that there are no clinically relevant differences in the results obtained depending on the harvesting technique used [25, 28]. The quadriceps graft, just like any other autograft, also has its disadvantages and is associated with morbidity, regardless of whether it is augmented by a bone block or not. Such factors must be considered when determining which graft is most suitable for a given patient profile and activity level [27]. The loss of extension strength after surgery is one of the most prominent concerns as it may affect the recovery and return to the pre‐injury level of competitiveness, especially in sports such as football [13].

In this context, isokinetic evaluations can objectively assess and address strength recovery and provide essential information concerning strength recovery patterns. This study aims to evaluate strength recovery, functional and clinical outcomes, and the associated morbidity of quadriceps tendon grafts with and without bone block in ACLR in football players.

## METHODS

### Participants

**Figure 1:**
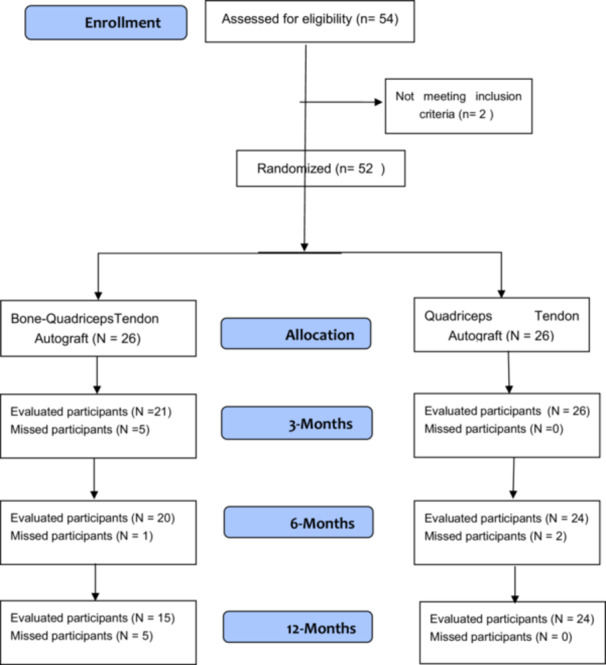
Flowchart of the study participants: The flowchart illustrates the participant follow‐up process in the study. A total of 54 patients were assessed for eligibility after exclusion, and a total of 52 participants were enroled. During the study, 11 participants missed the follow‐up.

This study included 52 competitive Spanish federal soccer players with complete ACL injuries, who were categorized into two groups: ACLR with the Bone‐Quadriceps Tendon Autograft (BQTA) and ACLR with the QTA, each comprising 26 participants (Figure 1). This study was approved by the local Biomedical Investigation Ethics Committee (Ref: LCA‐V2) and adhered to the Declaration of Helsinki. Informed consent was obtained from all participants before enrolment.

Eligibility criteria for participants were as follows: (1) a maximum of 6 months between the diagnosis of ACL injury and surgery, (2) a minimum of 3 years as a competitive soccer player and (3) an age between 16 and 40 years. The exclusion criteria were as follows: (1) history of joint injury or prior surgery on the affected knee, (2) any musculoskeletal injury within 4 weeks before surgery or untreated chronic injuries, (3) a period exceeding 6 months between injury and surgery, (4) removal of over 50% of either the lateral or medial meniscus during surgery and (5) articular cartilage lesions exceeding grade II on the Outerbridge scale.

### Sample size calculation

To determine the required sample size, this study established parameters with an alpha error of 5%. It aimed for a statistical power of 95%, specifically focusing on isokinetic knee extension tests conducted at 180/°s. Using these parameters, it was determined that a minimum of 22 participants per group was necessary. These calculations were performed using G*Power 3.1. Initially, the study enroled 52 patients and distributed them across two groups (BQTA and QTA), with 26 participants allocated to each group to accommodate an anticipated dropout rate of 20%.

### Group allocation and blinding methods

Following randomization, the assigned surgical technique (BQTA or QTA) was communicated to the surgeon and the respective patient, although patients remained unaware of the study hypothesis. Evaluators and data analysts were blinded to group assignments throughout the entire study period to prevent bias in outcome assessment and data analysis.

Baseline comparability between groups was assessed using the Chi‐square test for categorical variables, while normality and homogeneity of continuous variables were examined using the Shapiro–Wilk and Levene's tests, respectively. Following this assignment, the surgeon received group information for each patient, and the patients were informed about the type of graft that would be used in their surgery. To ensure the integrity of the study, the hypothesis was not disclosed to patients. Additionally, evaluators and data analysts were unaware of the group assignments throughout all phases of the study, ensuring an unbiased approach in both the implementation and analysis of the study results.

### Outcomes

The evaluation process of this study was structured into three distinct sections to assess the post‐surgery outcomes comprehensively. The first section involved patient‐related outcome measures (PROMs), assessed using the Lysholm Knee Scale (LKS) [1, 17]. The second section consisted of clinician‐reported outcome measures. These included measurements of thigh muscle girth, defined as the circumference of the thigh measured when the participant stood with separate legs and the body weight was evenly distributed. This measurement was performed using a 1.5‐m non‐elastic fibreglass cloth tape measure, which showed high reliability (ICC = 0.92) [2].

The third section, functional outcome measures (FROMs), assessed muscle strength through isokinetic dynamometry using the Genu 3 dynamometer. This evaluation measured hamstring and quadriceps muscle strength across various angular velocities, with tests conducted at 60°/s, 180°/s and 300°/s, incorporating a 1‐min rest between each set. The reliability of isokinetic dynamometry is well established (ICC = 0.88) [7]. Additionally, the single Limb Symmetry Index (LSI) was calculated to assess the peak torque (PT), further emphasizing the precision of this instrument.

Moreover, the single leg hop test (SLHT) was included in the FROMs to evaluate knee dynamic stability. Participants were instructed to jump forward as far as possible on a single leg and land firmly without losing balance. The distance jumped was measured from the starting line to the heel of the landing foot. The test was repeated three times, taking the highest score recorded and adhering to strict criteria that disqualified any attempt where balance was lost, additional hops were made after landing, or the non‐jumping leg touched down prematurely. The results were then analyzed for each side, and the LSI was recalculated, confirming the reliability of the test (ICC = 0.94) [6]. All participating patients were evaluated before surgery and again at 3, 6, 12 and 24 months after surgery. The participants were asked to attend a face‐to‐face assessment. The evaluation of endpoints was performed before the surgery and at 3, 6 and 12 months for all variables except for the PROMs and re‐injury rates, which were evaluated at 3, 6 and 12 months of follow‐up. All the measurements were assessed by an expert researcher with over 10 years of experience.

FROMs were performed after a 10‐min warm‐up session on a stationary bicycle, beginning with assessments on the non‐injured side. The initial preoperative evaluation was scheduled to be placed 1 week before the surgical procedure.

### Surgical intervention

The surgical procedure commenced with standard knee arthroscopy through an anterolateral portal to confirm the ACL injury and to identify any associated injuries, such as cartilage damage or meniscal tears. Subsequently, a low medial portal was established slightly above the joint line and 2 cm medial to the patellar tendon for surgical access. A high medial portal was created to enhance the visualization of the femoral footprint.

For the BQTA, a trapezoidal bone block measuring 20–25 mm in length and 7 mm in thickness was extracted from the proximal segment of the patella. Subsequently, the tendinous portion of the graft was carefully harvested using a scalpel and scissors to achieve a length of 8 cm. The tibial tunnel was established with a tibial guide laced in the centre of the anatomical ACL footprint medial to the posterior border of the anterior horn of the lateral meniscus and drilled with a cannulated drill of the same diameter as the graft. For the femoral tunnel, a K wire was placed in the centre of the anatomical femoral footprint below the lateral intercondylar ridge and the centre of the bifurcated ridge [8].

The K wire was drilled with the knee held in 120° of flexion. Subsequently, the tunnel was drilled.

The QTA involved a partial thickness of the quadriceps tendon that was 70–80 mm long, 7 mm deep and 10 mm wide, starting from the proximal edge of the patella and extending proximally to achieve a length of 8 cm. Graft fixation involved securing the graft in the femoral tunnel with a bioabsorbable interference screw measuring 20 mm in length and 9 mm in diameter (Biosure Regenesorb, Smith and Nephew Inc.). On the tibial side, the same fixation was achieved with a 10 mm diameter screw at 20° of knee flexion [4].

### Rehabilitation Protocol

Both groups adhered to an identical rehabilitation programme, which was conducted under the guidance of a physical therapist who was unaware of the patients' group assignments and the specifics of the study. The programme was structured into 6 distinct phases, each with defined criteria to be met before progressing to the subsequent phase. The details of each phase, along with the progression criteria, are outlined in Table 1.

**Table 1:** The pre‐ and post‐surgical rehabilitation programme.

Phases	Weeks	Objective	Treatment	Milestones
Presurgical rehabilitation	4 weeks	Full knee extension ROM Minimal knee effusion Full‐extension during straight leg raise	Knee flexor strength exercise. Knee extensor strength exercise.	Patients with full knee extension range of motion (ROM), absent or minimal effusion, and no knee extension lag during a straight leg raise preoperatively have better postsurgical outcomes, such as returning to previous levels of activity and demonstration of normal knee function.
Intermediate postoperative phase	Week 1	Full knee extension 90° of knee flexion Patellar mobilization To control pain and inflammation	Neuromuscular electrical stimulation. Knee patellar mobilization. Gait training. Stationary bike exercise. Home training programme: supine wall slides, self‐patellar mobilizations 30–50 times per day, quadriceps exercise and straight leg raise 3 times a day (3 × 10).	Active and passive ROM up to 90 knee flexion. Quadriceps contraction with patellar extension.
Early postoperative phase	Week 2	To achieve full ROM To activate knee muscles To control knee effusion To obtain a normal gait mechanism	ROM exercise through the pain‐free range. Incision mobilization after skin healed. Wall squats. Functional brace if swelling allows. Prone hangs. Knee Patellar mobilization.	Active knee flexion up to 110. Walking with a full extended knee and free of crutches. Stair reciprocal claiming. SLR without knee extension. The patient‐reported outcome of more than 65% in the overall evaluation.
Intermediate postoperative phase	Weeks 3–5	Muscle endurance To achieve full ROM To improve muscle strength To control knee effusion To obtain a normal gait mechanism	Tibiofemoral mobilizations if joint mobility is limited. Achieve more than 10 min per day of bike. Start with balance and proprioceptive tasks.	Achieve knee flexion with only 10 differences with the uninvolved side. Achieve more than 60% of LSI‐quadriceps muscle strength.
Late postoperative phase	Weeks 6–8	To progress the athlete to full unilateral weight‐bearing activities through improving strength training and work capacity	Progress all exercises in intensity and duration. Start running progression. Move to a fitness facility when all milestones are achieved.	Achieve more than 80% of LSI‐quadriceps muscle strength. Achieve normal gait. Achieve full ROM and similar to the non‐injured side. Knee effusion of trace or less.
Transitional phase	Weeks 9–12	To improve high athletic abilities in multi‐direction running and sport‐specific tasks based on sport type	Sports‐specific ‘football’ tasks. Agility training.	The patient reported an outcome of more than 70%. Achieve more than 85% of SLHTs. Achieve more than 85% of LSI‐quadriceps muscle strength.
Follow‐up functional and function testing	From 4 to 12 months	To improve high athletic abilities in multi‐direction running and other specific tasks based on sport type	Increase the progression of transition phase exercise. Emphasise single‐leg exercise and progress in the plyometric exercise	The patient reported an outcome of more than 70%. Achieve more than 85% of SLHT. Achieve more than 85% of LSI‐quadriceps muscle strength.

Abbreviations: LSI, Limb Symmetry Index; ROM, range of motion; SLHT, single leg hop test.

### Statistical analysis

Descriptive statistics were used for all variables, with continuous data as mean ± standard deviation and categorical data as percentages (%). The normality of data distribution was assessed using the Shapiro–Wilk test, while Levene's test evaluated the homogeneity of variances. The Chi‐square test was used to compare the baseline variables between the groups. A repeated measures analysis of variance (ANOVA) was used to examine the outcome variables (LKS, muscle girth measurements, Visual Analogue Scale [VAS], muscle strength and SLHT), with groups (BQTA and QTA) as the between‐subjects factor, and time points (pre‐surgery, 3‐, 6‐ and 12‐month post‐surgery) as the within‐subjects factor. Post hoc analyses with Bonferroni correction were performed for pairwise comparisons. Additionally, multiple imputation was used for missing data, creating five parallel databases for analysis, assuming that the data were Missing Completely at Random [35]. Statistical significance was set at *p* < 0.05, and all analyses were conducted using the IBM SPSS Statistics version 25.

## RESULTS

### Sociodemographic and clinical characteristics

The study initially enroled 54 patients for eligibility; 2 patients did not meet the inclusion criteria, resulting in a total of 52 patients with ACL injuries being randomized equally into two groups: BQTA and QTA with ACL injuries, 26 in the BQTA group and 26 in the QTA group. At the 3‐month follow‐ups, the BQTA group had 21 evaluated participants, with five missing participants. The reasons for exclusion in the BQTA group included two patients who sustained a patellar fracture and one patient who sustained a quadriceps avulsion.

All these complications occurred during the first 2 months after the surgery, and there were two disruptions due to COVID‐19. While the QTA group had all 26 participants with no missing.

By the 6‐month follow‐up, the BQTA group had evaluated 20 participants, with one additional missed participant, while the QTA group had evaluated 24 participants, with 2 participants who had missed.

Finally, at the 12‐month follow‐up, the BQTA group had 15 evaluated participants with five additional missed ones, while the QTA group maintained 24 evaluated participants with no missing in this follow‐up phase. One patient left at 6 months, and five patients left at 12 months in the BQTA group because they either relocated to a different place or did not answer the phone.

Two patients in the QTA group did not attend the 6‐month follow‐up because they did not wish to continue participating in the study. A comparative analysis of the demographic and clinical characteristics revealed no significant differences between the two groups, except in terms of education level (*p* = 0.02) and incidence of meniscal injury (*p* = 0.04), as outlined in Table 2.

**Table 2:** Sociodemographic and clinical characteristics of the participants.

	BQTA (26)	QTA (26)	Total	*p* value
Age	22.5 ± 4.6	18.7 ± 2.4	20.3 ± 3.7	0.08
Sex				
Male	23 (88.5)	23 (88.5)	66 (84.6)	1.0
Female	3 (11.5)	3 (11.5)	12 (15.4)	
Civil status				
Single	25 (96.2)	26 (100)	77 (98.7)	0.31
Married	1 (3.8)	0 (0)	1 (1.3)	
Education level				
Basic	10 (38.5)	8 (30.8)	26 (33.33)	**0.02**
Medal	5 (19.2)	14 (45.2)	31 (39.7)	
High	11 (42.3)	4 (15.4)	21 (26.9)	
Smoking				
Not smoker	21 (80.8)	26 (100)	71 (91.0)	0.019
Smoker	5 (19.2)	0 (0)	7 (9.0)	
Alcohol consumption				
No alcohol	12 (46.2)	13 (50)	41 (52.6)	0.70
Monthly	12 (46.2)	11 (42.3)	30 (38.5)	
Weekly	1 (3.8)	2 (7.7)	5 (6.4)	
Daily	1 (3.8)	0 (0)	2 (2.6)	
Weight (kg)	75.5 ± 13.1	69.0 ± 9.2	71.8 ± 12.3	0.14
Height (m)	172.4 ± 8.5	171.9 ± 6.1	169.9 ± 16.9	0.06
BMI (kg/m^2^)	25.1 ± 3.5	22.8 ± 2.3	24 ± 3.3	0.15
Fat percentage	19.2 ± 5.1	18.6 ± 7.2	19.1 ± 7.1	0.50
Muscle percentage	34.8 ± 4.5	32.7 ± 4.1	33.2 ± 5.2	0.10
Injured side				
Left	13 (50)	14 (53.8)	38 (48.7)	0.78
Right	13 (50)	12 (46.2)	40 (51.3)	
Dominant side				
Left	9 (34.6)	4 (15.4)	19 (24.4)	0.11
Right	17 (65.4)	22 (84.6)	59 (75.6)	
Competition level				
Professional	6 (23.1)	3 (11.5)	9 (11.5)	0.35
Federal	20 (76.9)	22 (84.6)	67 (85.9)	
Other	0 (0)	1 (3.8)	2 (2.6)	
Meniscus injury				
No	12 (46.2)	5 (19.2)	28 (35.9)	0.04
Yes	14 (53.8)	21 (80.8)	50 (64.1)	
Crutches (III day)*				
No	11 (50.0)	7 (26.9)	23 (31.1)	0.1
Yes	11 (50.0)	19 (73.1)	51 (68.9)	
Bike (III week)*				
No	12 (42.5)	10 (38.5)	32 (43.2)	0.26
Yes	10 (45.5)	16 (61.5)	42 (56.8)	
Running (III month)*				
No	10 (45.5)	4 (15.4)	23 (31.1)	0.02
Yes	12 (54.5)	22 (82.6)	51 (68.9)	
Return to normal activity**				
No	0 (0)	2 (9.1)	3 (5.5)	0.28
Yes	12 (100)	20 (90.9)	52 (94.5)	
Return to pre‐injury level***				
No	6 (50)	10 (45.5)	25 (45.5)	0.80
Yes	6 (50)	12 (54.5)	30 (54.5)	

*Note*: Data are shown as mean ± SD or mean (frequency).

Abbreviations: BQTA, Bone Quadriceps Tendon Autograft; kg, kilograms; kg/m^2^, kilograms/metres square; kPa, kilopascal; QTA, Quadriceps Tendon Autograft; SD, standard deviation.

* *N* = 21 BQTA and *N* = 26 QTA.

** *N* = 20 BQTA and *N* = 24 QTA.

*** *N* = 15 BQTA and *N* = 24 QTA.

**Figure 2:**
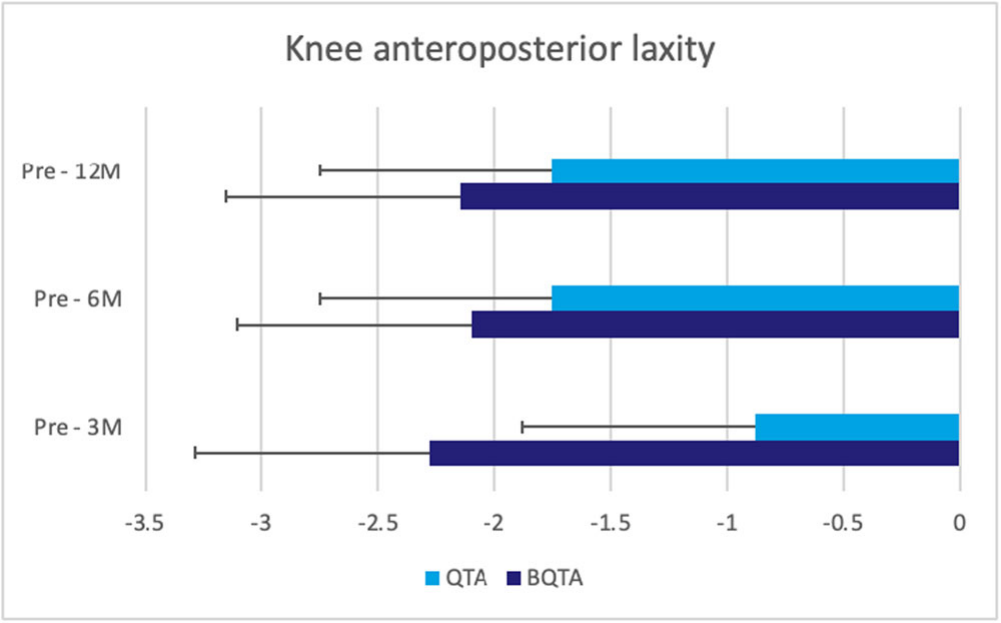
The figure illustrates the changes in knee anteroposterior laxity at 3‐, 6‐ and 12‐month follow‐up. The analysis shows a non‐significant trend (*F* = 2.26, *p* = 0.09, *ηp* ^2^ = 0.13) for the injured knee. The unit of measurement is presented in millimetres (mm). Statistical values represent the comparison of knee anteroposterior laxity at different points in time. BQTA, Bone‐Quadriceps Tendon Autograft; QTA, Quadriceps Tendon Autograft.

**Figure 3:**
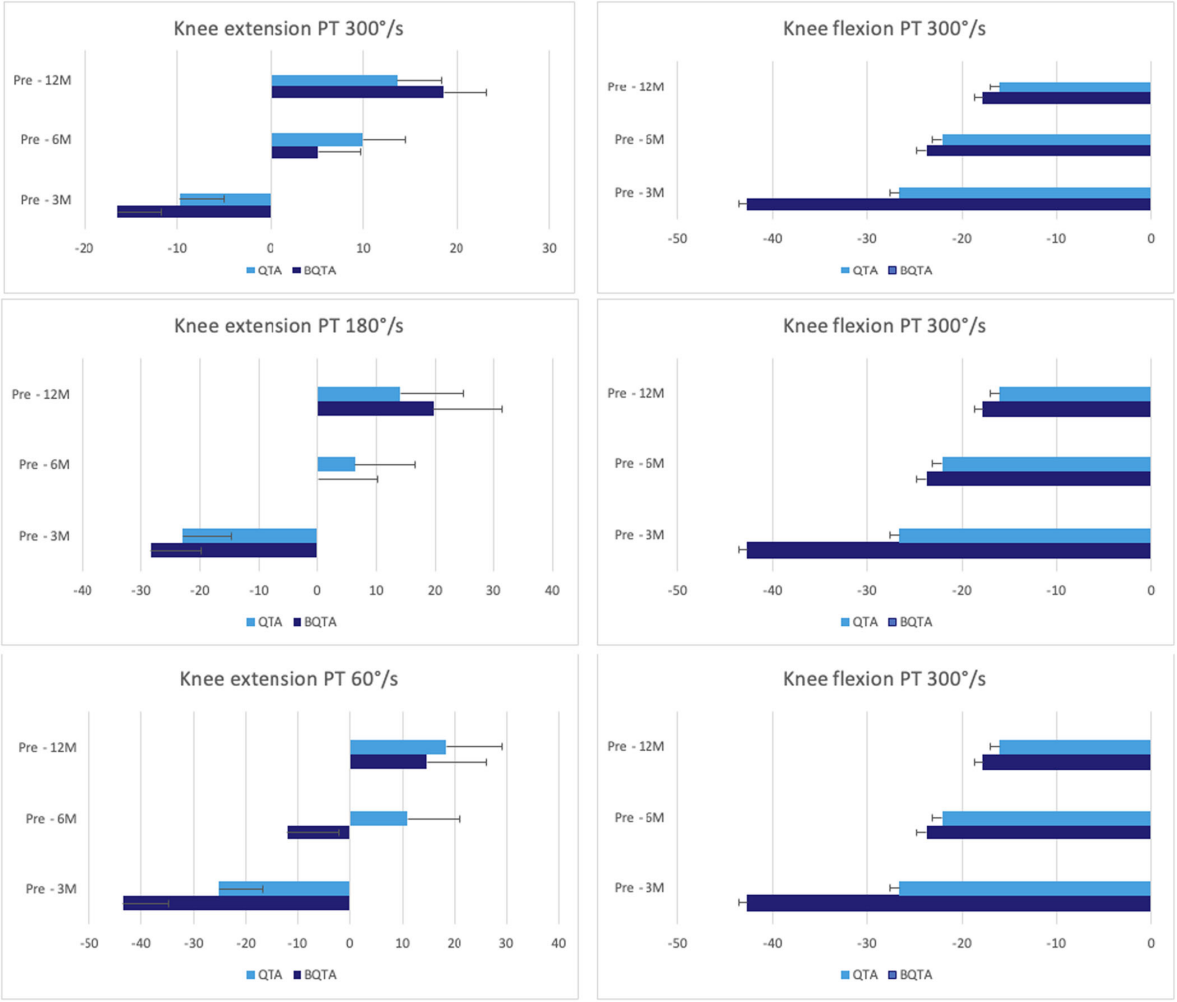
The figure illustrates the peak torque (PT) measurements of knee extension and flexion for the injured side at 3‐, 6‐ and 12‐month follow‐up time points for the QTA and BQTA groups. The PT values are presented at three angular velocities. 300°/s, 180°/s and 60°/s for knee extension and 300°/s for knee flexion. The PT is measured in Newton metre (Nm). The presented data for each follow‐up time point shows changes in muscle strength over time. The presented data highlights the changes in muscle strength and performance over time within the QTA and BQTA groups. BQTA, Bone‐Quadriceps Tendon Autograft; QTA, Quadriceps Tendon Autograft.

**Figure 4:**
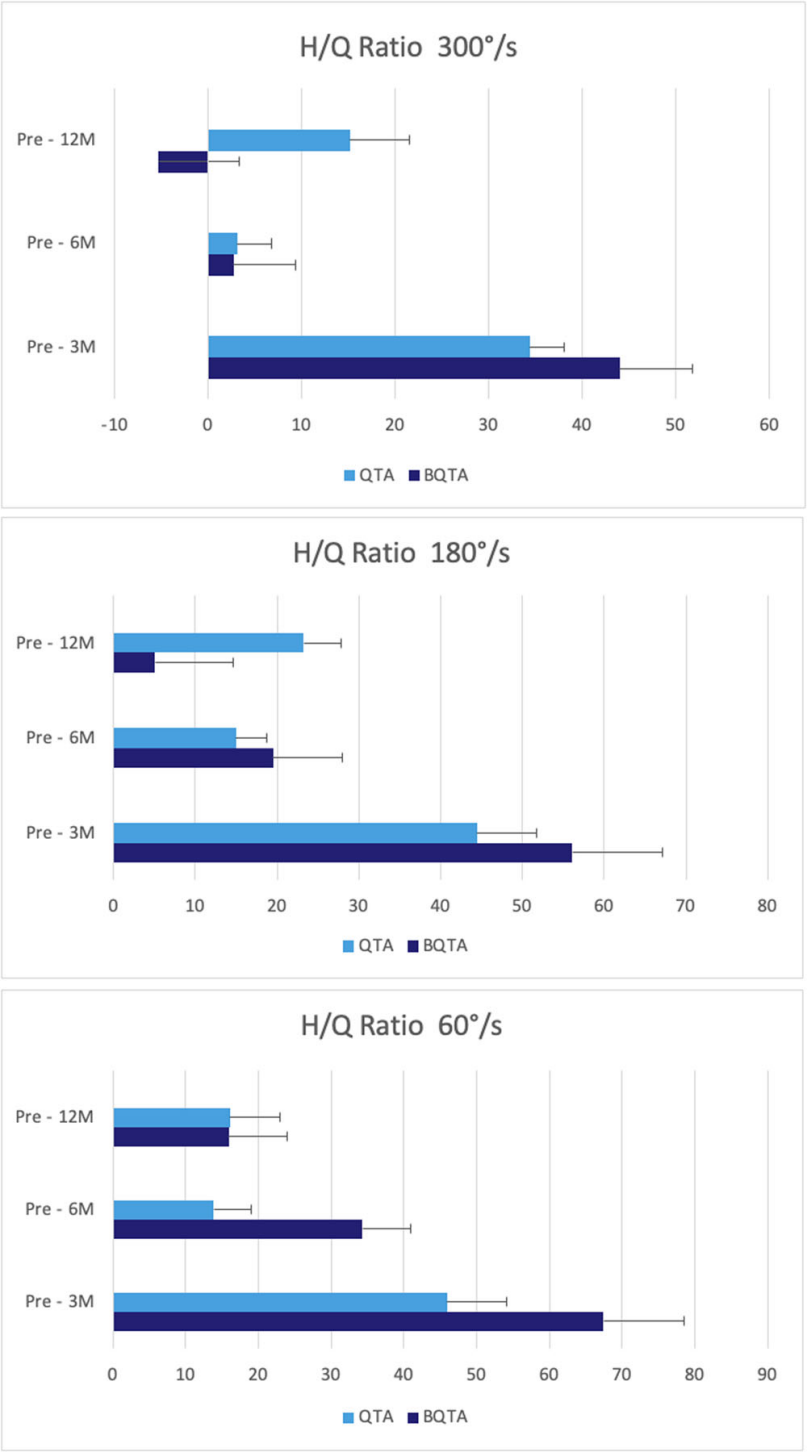
This figure illustrates the hamstring to quadriceps (H/Q) ratio of the injured side at 3‐, 6‐ and 12‐month follow‐up time points for the QTA and BQTA groups. The ratio is measured at three angular velocities: 300°/s, 180°/s and 60°/s, the relative strength between the hamstring and quadriceps muscles. The data are presented in Newton metre (Nm). Statistical analyses are included to assess changes in the H/Q ratio over time within the two groups. providing understanding of muscle recovery and balance during rehabilitation. BQTA, Bone‐Quadriceps Tendon Autograft; QTA, Quadriceps Tendon Autograft.

**Figure 5:**
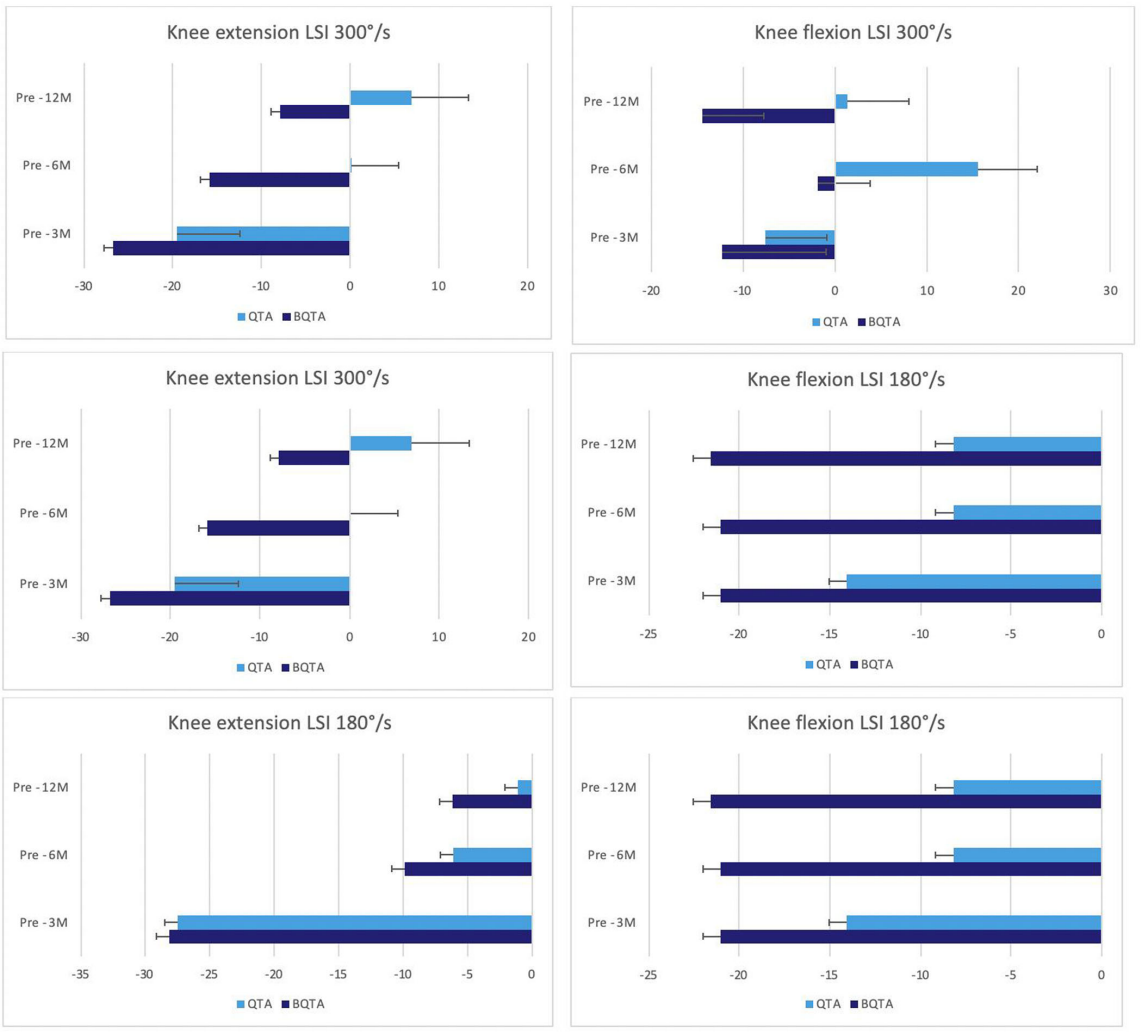
The figure illustrates the Limb Symmetry Index (LSI) of knee extension and flexion at 3‐, 6‐ and 12‐month follow‐up for the QTA and BQTA groups. The LSI values are presented at two angular velocities: 300°/s, 180°/s, for both knee extension and flexion, the LSI calculated to observe the symmetry between the injured and uninjured sides. The measurement unit is Newton metre (Nm). BQTA, Bone‐Quadriceps Tendon Autograft; QTA, Quadriceps Tendon Autograft.

**Figure 6:**
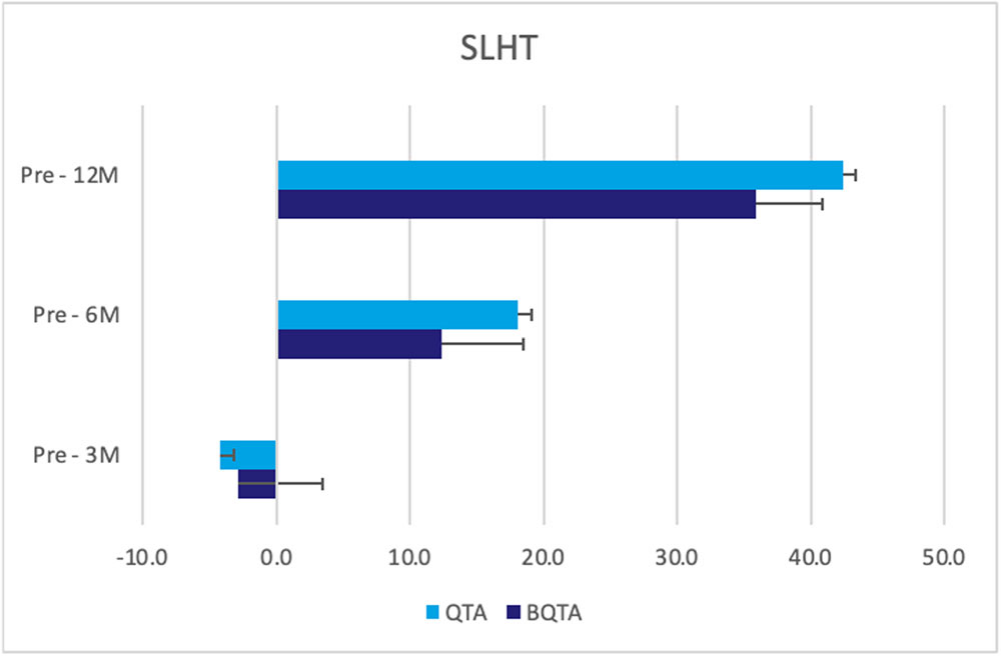
This figure illustrates the performance on the single leg hop test (SLHT) of injured leg at 3‐, 6‐ and 12‐month follow‐up for the QTA and BQTA groups. The test measures the ability to hop on the injured leg, providing an assessment of functional recovery and strength over time. The unit of measurement is centimetre (cm). BQTA, Bone‐Quadriceps Tendon Autograft; QTA, Quadriceps Tendon Autograft.

The repeated ANOVA of the LKS over time × autograft type did not show any significant differences for LKS (*F* = 1.54, *p* = 0.21, *ηp* ^2^ = 0.09) between experimental groups at 3, 6, 12 and 24 months of follow‐up (Table 3). During the follow‐up, two patients in the QTA group presented with graft tears, whereas one patient in the BQTA group presented with graft tears (Table 4). Regarding the knee anteroposterior laxity, the analysis of interaction time × autograft type did not display any significant results between groups (*F* = 2.26, *p* = 0.09, *ηp* ^2^ = 0.13) for the injured knee (Figure 2). The repeated ANOVA analysis of VAS, PT for knee flexion and extension at different speeds, hamstring/quadriceps (H/Q) ratios, LSI and SLHT—revealed no statistically significant differences (*p* < 0.05) at 3‐, 6‐ and 12‐month of follow‐up between autograft types (Figures 3, 4, 5, 6). The VAS was used to measure pain while resting (Table 5).

**Table 3:** Patient‐reported outcome measures at 3‐, 6‐ and 12‐month follow‐up.

Autograft type	Pre – 3 months evaluation	Pre – 6 months evaluation	Pre – 12 months evaluation
LKS			
BQTA	−0.28 ± 24.17 (−10.73 to 10.17)	14.46 ± 17.2 (7.2–21.72)	16.56 ± 14.67 (10.21–22.9)
QTA	6.36 ± 15.2 (0.22–12.49)	12.08 ± 15.6 (5.77–18.38)	11.14 ± 11.96 (6.31–15.97)

*Note*: This table presents patient‐reported outcome measures between baseline measurement and 3, 6 and 12 months of follow‐up.

**Table 4:** Re‐rupture rate for the patient.

Groups	Number of patients/re‐ruptures	Rupture rate
QTA	26/2	7.6%
BQTA	26/1	3.8%

*Note*: This table presents the number of re‐ruptures of the patient and the calculated rupture rate.

Abbreviations: BQTA, Bone‐Quadriceps Tendon Autograft; QTA, Quadriceps Tendon Autograft.

**Table 5:** Thigh girth injured side and Visual Analogue Scale (VAS) at 3‐, 6‐ and 12‐month follow‐up.

Autograft type	Pre – 3 months evaluation	Pre – 6 months evaluation	Pre – 12 months evaluation
Thigh girth injured side (cm)			
BQTA	−1.31 ± 2.61 (−2.44 to −0.18)	−0.6 ± 4.05 (−2.35 to 1.15)	0.11 ± 4.62 (−1.89 to 2.11)
QTA	−0.35 ± 2.59 (−1.4 to 0.69)	0.37 ± 2.99 (−0.84 to 1.58)	1.56 ± 3.98 (−0.04 to 3.17)
VAS			
BQTA	−0.86 ± 2.64 (−1.93 to 0.2)	−1.06 ± 2.26 (−1.97 to −0.14)	−1.29 ± 2.13 (−2.15 to −0.42)
QTA	−0.75 ± 1.98 (−1.55 to 0.05)	−1.27 ± 2.25 (−2.18 to −0.36)	−1.69 ± 1.85 (−2.44 to −0.95)

*Note*: This table presents the measurements of thigh girth (injured side) and the VAS. The numbers represent the differences from the baseline pre‐surgical condition (Pre) at three different points: from baseline to 3 months, from baseline to 6 months, and from baseline to 12 months for BQTA and QTA groups. Data are shown as mean ± SD (95% CI) and measured by repeated ANCOVA test, *p* < 0.05. The thigh girth measurements are provided in centimetres (cm), while the VAS scores are presented as changes in pain perception.

Abbreviations: BQTA, Bone‐Quadriceps Tendon Autograft; QTA, Quadriceps Tendon Autograft; SD, standard deviation.

## DISCUSSION

This study assessed the clinical and functional outcomes of ACLR using two different QTA techniques: BQTA and QTA. A total of 52 patients with ACL injuries were evaluated. No significant differences were observed in the outcomes at the final follow‐up. Key metrics such as the LKS (mean difference = 1.2, 95% CI: [−0.8, 3.2], *p* = 0.21), VAS for pain (mean difference = 0.3, 95% CI: [−0.5, 1.1], *p* = 0.68), PT, H/Q ratios and LSI showed no significant differences between the groups. However, due to complications, the final analysis was conducted with 48 participants (22 in the BQTA group and 26 in the QTA group). The comparative analysis between the groups across various metrics—LKS, knee anteroposterior laxity, re‐rupture rate, VAS for pain, PT for knee flexion and extension at different speeds, H/Q ratios, LSI and SLHT—revealed no statistically significant differences (*p* < 0.05). Aside from variations in education level and the presence of meniscal injuries, the baseline parameters were nearly identical between the groups.

Furthermore, we have mentioned that the BQTA and QTA groups share a very similar situation. An additional biomechanical investigation from Straus et al. [31] showed that a quadriceps graft with bone block seems to be the best option from a biomechanical point of view. Moreover, harvesting the quadriceps tendon with a bone block could enhance graft maturation. However, a systematic review by Jackson et al. [15] found no difference in the re‐rupture rate between QTA and BQTA. Besides the mentioned results of all studies, this heterogeneity of the graft itself under the name ‘quadricipital graft’ could cause a bias in the interpretation of clinical results since there are significant biomechanical differences depending on the option of the quadriceps tendon of those previously mentioned [5].

Our study did not show any difference in VAS between the two groups, However, the previous review mentioned found a higher rate of anterior knee pain in patients who underwent surgery without a bone block than in those with a bone block [15]. This may seem counterintuitive, and the authors suggested that factors beyond graft harvesting may contribute to anterior knee pain. Crum et al. [5], in another systematic review, found no differences in objective and patient‐reported outcomes but did find a higher incidence of complications in patients operated on with a bone block. In a retrospective study analyzing 708 patients undergoing ACLR with and without a bone block, Setliff et al. [28] found no differences in International Knee Documentation Committee Subjective outcomes, re‐rupture rates or complication percentages. Similarly, Runer et al. [25] found no differences in patient‐reported outcome measurements or subsequent surgical interventions between patients who underwent surgery with or without a bone block. In a recent systematic review, Meena found no differences in clinical outcomes, re‐rupture rate or complications, although the 1.2% overall patellar fracture rate only occurred in patients with BQTA. Lind and Nielsen recently published the results of the Danish ligament registry. They analyzed 1500 patients with 7 years of overall follow‐up and concluded that both grafts exhibited comparable revision rates and sagittal knee stability, but BQTA achieved better rotational stability with less pivot shift than QTB [16].

Although harvesting a quadriceps tendon graft with a bone block is associated with a higher risk of patellar fracture, it may sometimes be necessary when the quadriceps tendon length is less than 60 mm. This potentially devastating complication is four times more frequent in patients who undergo quadriceps harvesting with a bone block than in those with a Bone‐Patellar Tendon‐Bone graft [10, 32, 34]. Fu et al. [9] published a series of 57 patients who underwent quadriceps tendon grafting with a bone block and observed a patellar fracture rate of 8.8%. They reviewed the factors associated with an increased risk of patellar fractures and recommended guidelines to minimize this risk. These guidelines include harvesting the bone graft from a centralized location, limiting the bone graft defect to the superior pole of the patella, ensuring that the depth of cut is less than 50% of the patellar thickness, keeping the length of the bone block less than 50% of the patellar length, and creating a trapezoidal bone block that is wider anteriorly than posteriorly [9]. Aligned with the recommendations to reduce the risk of fracture following quadriceps tendon graft harvesting, Perry et al. [23] concluded from a cadaveric study that extracting the bone block from the lateral part of the patella increased the risk of fracture. Based on MRI research, Negrin et al. [20] recommended that the bone block be excised from the medial half of the central area, with the outer edge of the saw positioned at the medial border of the central area. A graft measuring 15 mm in length, 10 mm in width and 8 mm in depth can be safely harvested from all white male participants, and almost all female participants are taller than 165 cm.

One limitation of the study was the sample size; however, the results of the isokinetic assessment were statistically not significant. The recovery of strength following graft extraction in ACLR is a crucial aspect related to the morbidity of the donor site and can influence both the rehabilitation process and surgical outcome [14, 36]. Previous research has highlighted superior strength recovery with quadriceps tendon grafts compared with hamstring or patellar tendon grafts [11, 19, 33]. However, to our knowledge, this study is the only one to investigate this variable in patients operated on by the same surgeon using a quadriceps tendon graft with and without a bone block. Another limitation is that the rate of meniscus injuries in the analysis has not been controlled, given that 81% of subjects in the QTA group had meniscus injuries; this may have influenced the study outcomes. Future studies should consider controlling meniscus injury rates to observe the effect better.

The results of our research indicated that there were no differences in the recovery of flexion or extension strength between the two groups, suggesting that strength recovery should not influence the decision when choosing between graft options. However, it is noteworthy that several serious complications occurred in the group that underwent surgery with a bone block, including two patellar fractures and one quadriceps tendon avulsion. The incidence of patellar fracture in this study is very similar (8.7%) to that reported by Fu et al. [9], previously mentioned. The depth of the bone block extraction during surgery was correct, but the recommendations based on the media‐lateral positioning of the patella, cited in this manuscript, were not considered, which may explain these complications. Although both cases involved a traumatic event, the first patient suffered a fall in the shower 2 weeks post‐operation, and the second was struck by a heavy object from a height onto the knee 1‐month post‐surgery. Another serious complication related to graft harvesting was the avulsion of the quadriceps tendon in a patient descending stairs 6 weeks after surgery, which, interestingly, though rare, has also been described in association with the extraction of a bone block [22].

Lastly, one of the study's strengths is the homogeneity of the patient sample, as it consisted of registered soccer players who were operated on by the same surgeon and followed the same rehabilitation protocol carried out by the same physiotherapist. Given these similarities, we believe that the observed complications occurred only in the group of patients who underwent surgery with a bone block. These findings can aid in making decisions about the necessity of incorporating a bone block during the extraction of the quadriceps tendon graft and, if so, to ensure that it is performed by the technical recommendations previously described.

## CONCLUSION

Similar functional and clinical results were observed with BQTA and QTA after ACLR in football players. Major complications were associated with the extraction of the bone block exclusively in the BQTA, which might guide the surgeon in choosing the type of quadriceps graft.

## AUTHOR CONTRIBUTIONS

Conceptualization: Jose Luis Martín Alguacil and Carolina Fernandez‐Lao. Methodology: Jose Luis Martín Alguacil and Fahed Herbwi. Formal analysis and investigation: Fahed Herbwi, Mario Lozano‐Lozano and Manuel Vides Fernánde. Writing—original draft preparation: Manuel Vides Fernánde and Fahed Herbawi. Writing—review and editing: Carolina Fernandez‐Lao and Mario Lozano‐Lozano. Supervision: Carolina Fernandez‐Lao, Mario Lozano‐Lozano and Juan Carlos Monllau.

## CONFLICT OF INTEREST STATEMENT

The authors declare no conflicts of interest.

## ETHICS STATEMENT

This study was approved by the local Biomedical Investigation Ethics Committee (Ref: LCA‐V2) and adhered to the Declaration of Helsinki. Informed consent was obtained from all participants before enrolment.

## CLINICAL TRIAL REGISTRATION

NCT04742868.

## Data Availability

The data supporting this study's findings are available upon request from the corresponding author.
